# Exploring Large Domain Motions in Proteins Using Atomistic Molecular Dynamics with Enhanced Conformational Sampling

**DOI:** 10.3390/ijms22010270

**Published:** 2020-12-29

**Authors:** Hisham M. Dokainish, Yuji Sugita

**Affiliations:** 1 RIKEN Cluster for Pioneering Research, 2-1 Hirosawa, Wako, Saitama 351-0198, Japan; hisham.dokainish@riken.jp; 2 RIKEN Center for Computational Science, Integrated Innovation Building 7F, 6-7-1 Minatojima-minamimachi, Chuo-ku, Kobe, Hyogo 650-0047, Japan; 3 RIKEN Center for Biosystems Dynamics Research, Integrated Innovation Building 7F, 6-7-1 Minatojima-minamimachi, Chuo-ku, Kobe, Hyogo 650-0047, Japan

**Keywords:** molecular dynamics, enhanced conformational sampling algorithm, ribose binding protein, gREST_SSCR, free energy landscapes, hinge and twist angles, inter-domain salt bridges

## Abstract

Conformational transitions in multidomain proteins are essential for biological functions. The Apo conformations are typically open and flexible, while the Holo states form more compact conformations stabilized by protein-ligand interactions. Unfortunately, the atomically detailed mechanisms for such open-closed conformational changes are difficult to be accessed experimentally as well as computationally. To simulate the transitions using atomistic molecular dynamics (MD) simulations, efficient conformational sampling algorithms are required. In this work, we propose a new approach based on generalized replica-exchange with solute tempering (gREST) for exploring the open-closed conformational changes in multidomain proteins. Wherein, selected surface charged residues in a target protein are defined as the solute region in gREST simulation and the solute temperatures are different in replicas and exchanged between them to enhance the domain motions. This approach is called gREST selected surface charged residues (gREST_SSCR) and is applied to the Apo and Holo states of ribose binding protein (RBP) in solution. The conformational spaces sampled with gREST_SSCR are much wider than those with the conventional MD, sampling open-closed conformational changes while maintaining RBP domains’ stability. The free-energy landscapes of RBP in the Apo and Holo states are drawn along with twist and hinge angles of the two moving domains. The inter-domain salt-bridges that are not observed in the experimental structures are also important in the intermediate states during the conformational changes.

## 1. Introduction

Large-scale conformational transitions in multidomain proteins play essential roles in numerous biological processes including allosteric regulation, signaling and catalysis [1,2,3,4]. The transitions from the inactive to active states often govern their biological functions [2,3,5]. In fact, multidomain proteins constitute more than two thirds of the proteome [4,6]. Unraveling conformational intermediates, transition states, pathways and energetics along the transition pathways are fundamental knowledge in biochemistry and molecular biology. This is also important to alter protein functions through interfering with conformational dynamics using drugs or small compounds [7,8]. Although structural information on multidomain proteins has been accumulated with X-ray crystallography, nuclear magnetic resonance (NMR) and cryo-electron microscopy, conformational dynamics of multidomain proteins are still difficult to describe in the atomic resolution [9,10,11,12]. Classical molecular dynamics (MD) simulation could characterize the conformational dynamics of proteins or other biomolecules, while it often fails to predict large-scale conformational transitions that happen in the milliseconds, or slower, time scales [13,14,15,16].

Conformational dynamics of multidomain proteins are often described as relative domain movements, where rigid structural units are defined as domains in the motions [17]. Such motions are typically described as hinge-bending, twisting and their combinations. Structural bioinformatics tools, for instance, HingeFind [18], DynDom or DynDom3D [19,20,21], have annotated various domain motions of proteins whose multiple structures are found in the protein data bank (PDB). The results are summarized in several structural databases [22,23]. Motion tree (MT) is a single tree diagram determined by a hierarchical clustering of local conformational changes [24,25], and is able to define rigid structural units and flexible regions from two known structures without a priori knowledge. Recently, we have used MT to give reasonable inter-domain contact interactions in Go-like coarse-grained (CG) model potential and succeeded to simulate large-scale domain motions of RBP and glutamine binding protein (GBP) [26]. In this study, we propose an alternative atomistic MD simulation method to study conformational dynamics of multidomain proteins with or without an explicitly bound ligand.

Enhanced conformational sampling algorithms, such as temperature replica-exchange MD (T-REMD), replica exchange with solute tempering (REST or REST2), Gaussian accelerated MD (GaMD) and so on, have often been employed to study slow dynamics of proteins and other biomolecules [27,28,29,30,31,32,33,34]. In REST/REST2, motions of a selected solute molecule are enhanced at higher solute temperatures, while the rest of the system is simulated at room temperature in all replicas [35,36,37,38]. Recently, Kamiya et al. introduced a more flexible selection of solute in a similar scheme to REST2 [39]. In this extension which is referred to as gREST (generalized REST), a part of the solute molecule with all or a part of the potential energy terms can be selected as the solute region. For instance, in protein-ligand binding simulations, not only a ligand molecule but also protein sidechain residues near the active site are simulated as “solute” in gREST, which can accelerate ligand binding or unbinding events significantly [39,40,41,42]. The framework of gREST allows us to select “solute” regions for target conformational motions of biomolecules to be investigated in detail. To enhance conformational dynamics of multidomain proteins, we propose to select only surface charged residues as the solute region in gREST simulations. This approach, which we refer to as gREST selected surface charged residues (gREST_SSCR), has two advantages compared to the conventional MD (cMD) and other enhanced sampling methods: (i) The intra-domain interactions remain intact, keeping the conformational stability of each domain even at high solute temperatures; only relative domain motions can be enhanced in gREST_SSCR. (ii) The number of atoms in the solute region is much smaller than conventional REST/REST2, reducing the number of replicas. This allows simulation of a very large biomolecule with reasonable computational resources.

We apply gREST_SSCR to investigate conformational transitions of the G134R mutant of ribose binding protein (RBP_G134R_), which is a member of the periplasmic binding protein (PBP) superfamily [43,44]. PBP functions as a soluble receptor for numerous ligands and plays important roles in nutrient uptake and bacterial chemotaxis [43]. PBP consists of two Rossman-fold domains and a hinge region (2-3 𝛽-strands) that connect the domains [45]. Atomic structures of several PBP members determined using X-ray crystallography show conformational transitions from the Apo_open to Holo_closed forms upon ligand binding [5,44,46,47,48,49,50,51]. However, molecular mechanisms underlying the open-to-closed transitions remain puzzling [10]. For instance, a computational/NMR study suggested that the Apo state of glutamate binding protein (GBP) takes close forms with a probability of 40% [52], while a more recent NMR study showed that it is highly improbable to take closed forms in the Apo state [10]. In contrast, the formation of a semi-closed state in the Apo state was shown for maltose and glucose/galactose binding proteins [11,45]. It is also unclear if conformational transitions in PBP are explained based on the induced fit or the conformational selection [53,54]. Because of the availability of multiple X-ray structures of both closed and open forms, and the small molecular size, RBP is considered as a reasonable target in this study. Atomistic MD simulations of RBP_G134R_ in the Apo and Holo states based on gREST_SSCR could extend their conformational spaces so that molecular mechanisms for the open-to-closed transitions have been investigated in detail.

## 2. Results

### 2.1. Structures of RBP_G134R_ in the Apo and Holo States

**Figure 1:**
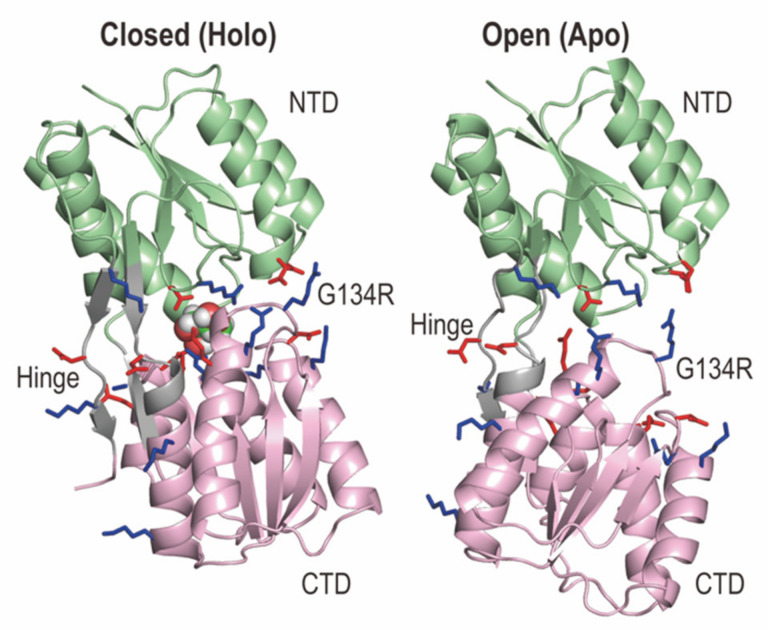
Structures of the G134R mutant of ribose binding protein (RBP_G134R_) in the Holo (left) and Apo (right) states. The Holo state takes a closed conformation with a bound ribose, whereas the Apo state shows an open structure consisting of the N-terminal (NTD in pale green) and C-terminal (CTD in light pink) domains. NTD is defined with the residues 1–100 and 236–259, while CTD is for the residues 108–231 and 269–271. The hinge region between NTD and CTD (residues 101–107, 232–235, 260–268) is shown in grey. The blue and red side chains are positively and negatively charged amino acids, which are selected as solute in gREST_SSCR. The mutant structure in Holo RBP was taken from the protein data bank (PDB ID: 1drj), while that in Apo RBP was modeled using chain A of the X-ray structure of wild type RBP (PDB: 1urp).

We used Apo and Holo (in complex with a ribose) structures of the G134R mutant of RBP (RBP_G134R_), because of three reasons: (i) The global structures of RBP_G134R_ are very similar to those of the wild type; the heavy atom root mean square deviation (RMSD) between the X-ray structures of wild type and the G134R mutant in the Holo state is 0.2 Å. (ii) The mutation increases the binding affinity to a ribose compared to a wild type [44]. (iii) There are two salt-bridge interactions (Asp67-Arg134 and Asp69-Arg134) in Holo RBP_G134R_, while there are no such interactions in both Apo and Holo states of wild type. We selected 22 out of 65 charged residues in RBP_G134R_ as the solute region in gREST_SSCR (Figure 1). Most of the selected charged residues are near the interface of the two domains, where few distant charged residues were also selected to ensure the neutrality of the solute. The number of atoms in the solute region is 393. gREST_SSCR simulations with 12 replicas were performed for 250 ns in Apo and Holo RBP_G134R_, respectively, while the cMD simulations were carried out for 2 μs in each state. We applied weak distance restraints between the center of mass (COM) of a ribose and the Cα atom of Ser103 of RBP_G134R_ in Holo simulations. Further details of simulation conditions are described in the Material and Methods section.

### 2.2. gREST_SSCR Simulations of RBP_G134R_ in the Apo and Holo States

#### 2.2.1. How gREST_SSCR Works in RBP_G134R_ Simulations

**Figure 2:**
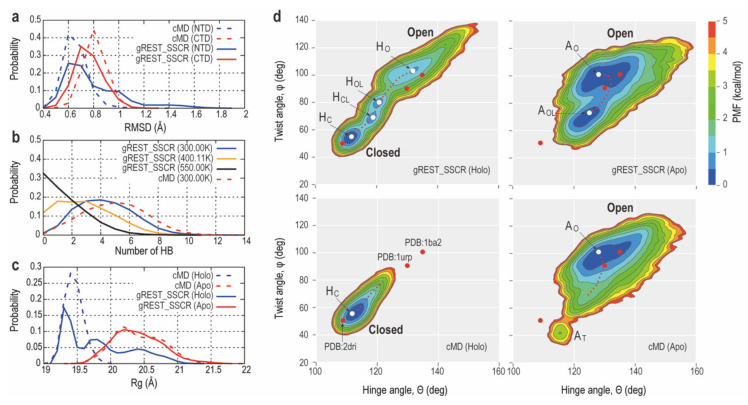
(**a**) Probability distributions of the Cα atoms root mean square deviation (RMSD) in CTD and NTD in conventional molecular dynamics (cMD) (dashed line) and gREST_SSCR Holo simulation (solid line) both at 300.00 K. RMSD of NTD and CTD are shown in blue and red, respectively. (**b**) Probability distributions of H-bonds in the Holo state between the 22 selected residues in the solute region of gREST_SSCR simulation at 300,00, 400.11 and 550.00 K (solute temperatures). As a reference, the same distribution obtained in cMD at 300.00 K is shown as a dotted line. (**c**) Probability distributions of radius of gyration, *Rg*, in cMD (dashed line) and gREST_SSCR (solid line) both at 300.00 K. The lines’ colors are blue and red for gREST_SSCR and cMD, respectively. (**d**) The free-energy landscapes obtained in cMD and gREST_SSCR simulations at 300.00 K (cMD (Holo): bottom left, cMD (Apo): bottom right, gREST_SSCR (Holo): Top left, and gREST_SSCR (Apo): Bottom left). Cluster centers are shown in white points (H_O_, H_OL_, H_CL_, H_C_, and A_O_, A_OL_, A_T_). Red points represent three PDB structures (2dri, 1urp and 1ba2).

We first examine how the gREST algorithm works properly in the simulations. In Figure S1, random walks of selected replicas in the solute temperature space are shown. We indeed observed good random walks between 300.00 and 550.00 K in the space. It becomes possible due to the sufficient overlaps of potential energies between replicas at neighboring solute temperatures (Figure S2). Next, the Cα atom root mean square deviations of NTD and CTD in Apo (Figure S3a) and Holo (Figure 2a) in RBP_G134R_ are compared between cMD and gREST_SSCR at 300.00 K. The N-terminal (NTD) and C-terminal (CTD) domain structures in gREST_SSCR at 300.00 K are almost equally stable compared to those in cMD at the same temperature. Since we selected only the surface charged residues as the solute region in gREST simulations and changed their temperatures in each replica, the results in the intra-domain conformational stability are reasonable. We also compare the number of H-bonds between the 22 selected charged residues in RBP_G134R_ at three solute temperatures (300.00, 400.00 and 550.00 K) in gREST_SSCR and in cMD at 300.00 K (Figure 2b for Holo and Figure S3b for Apo). The number of H-bonds at 300.00 K in gREST_SSCR is similar to that in cMD, while the number of H-bonds is greatly reduced as the solute temperature increases in gREST_SSCR. The trends are true both in Apo and Holo states, suggesting that the replicas at higher solute temperature in gREST_SSCR give more opportunities for relative domain motions.

#### 2.2.2. Comparison of Conformational Sampling Abilities between cMD and gREST_SSCR

Analysis of the radius of gyration, *Rg*, of RBP_G134R_ shows that neither of the 2 μs cMD simulations from Holo_closed nor Apo_open states were able to reach the opposite state (Figure 2c). Indeed, applying gREST_SSCR drastically enhanced conformational sampling wherein the Holo simulation was able to sample closed, open and intermediate states which are characterized by three distinct peaks in the *Rg* distribution plot (Figure 2c). To better quantify the open-closed transition, we examine the free-energy landscapes in Apo and Holo states observed in gREST_SSCR and cMD at the same temperature (300.00 K) in Figure 2d. The landscapes are described along with the two interdomain angles defined below: (1) Hinge angle (θ), which is the bending angle formed by the centers of mass (COMs) of NTD (residues: 1–100, 236–259) and CTD (residues: 108–231, 269–271) and the hinge region (residues: 101–107, 232–235, 260–268); (2) twist angle (φ), which is the dihedral angle formed by COMs of NTD and CTD as well as those of the base regions of NTD (residues 99–100, 236–237 and 258–259) and CTD (residues 108–109, 230–231 and 269–270). Both angles were previously used to describe the open-closed transitions in previous studies of PBP [45,47,49].

In Apo RBP_G134R_ (Figure 2d, right), the conformational space sampled using gREST_SSCR at 300.00 K is almost equal to that with cMD. However, the Apo_open_like form (A_OL_), whose twist angles (φ) are different from the Apo open form (A_O_) and cannot be obtained using cMD at 300.00 K. Instead, cMD simulation samples a distinct structure with twisting angle φ = 35–50 (deg), which we refer to as A_T_. This looks similar to the closed X-ray structure with a bound ribose. However, their hinge angles are different from each other. In Holo RBP_G134R_ (Figure 2d, left), we observe more drastic differences in the conformational spaces sampled with gREST_SSCR and cMD at the same temperature (300.00 K). cMD can sample only the closed conformations, while gREST_SSCR at 300.00 K gives us four distinct forms, namely, Holo_closed (H_C_), Holo_closed_like (H_CL_), Holo_open_like (H_OL_) and Holo_open (H_O_) forms. H_C_ and H_O_ correspond to structures having similar hinge and twist angles of X-ray crystal structures in Apo and Holo states, respectively. The transition from H_C_ to H_CL_ is characterized by changes in both twist (φ) and hinge angles (θ). H_CL_ and H_OL_ are different only in their twist angle (φ). From H_OL_ to H_O_, both twist (φ) and hinge angles (θ) are changed significantly. H_O_ shows a great flexibility, while its conformational space is not completely overlapped with A_O_.

#### 2.2.3. Intermediate Structures of RBP_G134R_ Stabilized by the Inter-Domain Salt-Bridge Interactions

**Figure 3:**
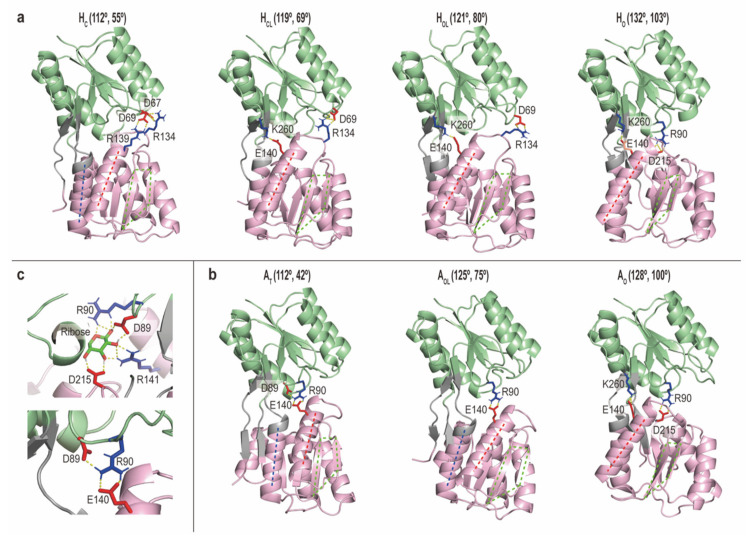
Representative structures of metastable states in Holo (**a**) and Apo (**b**) RBP_G134R_. (**c**) The sidechain interactions with a bound ribose (top) and those in Apo (A_T_) (bottom). Interdomain salt-bridge interactions are highlighted using stick representations. In (**a**,**b**), the representative hinge angles (θ) and twist (φ) are shown in parenthesis.

To characterize key interactions, the average salt-bridge interactions in each metastable state are shown in Figure S4. From the closed to open states, namely, in the order of H_C_, H_CL_, H_OL_ and H_O_, a gradual reduction of interactions between three main loop sites in NTD (residue 8–15, 38–45 and 66–70) and three loop sites in CTD (residue 129–138, 161–168 and 187–195) is observed. The key electrostatic interactions in each metastable state are summarized in Table 1 and Figure 3. Herein, H_C_ conformations are characterized by salt bridges between Asp67 and Arg134 or between Asp69 and Arg139. These interactions are observed in Holo cMD simulation as well as X-ray structure of Holo RBP_G134R_ (PDB:1drj). On the contrary, intermediate states (H_CL_ and H_OL_) show that Asp69 mainly interacts with Arg134. A transit salt bridge on the opposite side of the protein is formed between Glu140 and Lys260 in H_CL_, H_OL_ and H_O_. In A_O_ in gREST_SSCR and cMD, the interdomain salt-bridges between Arg90 and Asp215, and between Glu140 and Lys260, are the most dominant, while in A_OL_ and A_T_, Arg90 interacts with Glu140, which is a clear difference from the interactions in A_O_. Note that although that H_O_ and A_O_ are not fully overlapped, they both show the formation of similar salt bridges. A comparison of the interactions at fully closed states (H_C_ in gREST_SSCR and H_C_ in cMD) or fully open states (H_O_, A_O_ in gREST_SSCR and All in cMD (Apo)) show similar H-bonding patterns, reflecting that the observed interactions are not an artifact of solute selection.

In Figure 3, representative structures and interactions around the ligand binding sites are shown both in Apo and Holo states. It is interesting to see the switch of interaction partners in the open-closed transition: for instance, the interaction in Arg90 and Asp215 is observed both in H_O_ and A_O_, while both sidechains are important to bind a ribose in H_C_. In A_T_, Asp89 and Arg90 are involved in the interdomain salt-bridge interaction with Glu140, which are not observed in the closed form. It suggests that A_T_ is not functionally relevant compared to the other metastable states.

## 3. Discussion

### 3.1. How gREST_SSCR Can Enhance Conformational Sampling of Large-Scale Domain Motions of Proteins

In this study, we propose a simple but powerful conformational sampling scheme, which we call gREST_SSCR, for large-scale conformational changes in multi-domain proteins. This method is based on the framework of REST2 or gREST. However, we can fully utilize the advantage of gREST over the conventional REST or REST2, which is the flexible selection of the solute region in this algorithm. In gREST_SSCR, only selected surface charged residues in a multidomain protein are defined as the solute region to enhance conformational dynamics. This allows intact intra-domain conformational stability which enhances the relative motions of multiple domains as we see in the simulations of Apo and Holo RBP_G134R_. Note that gREST_SSCR is considered a very “mild” enhanced conformational sampling scheme, since we can keep most of intra- and inter molecular interactions intact even in the replica simulated at higher solute temperatures. Only the Lennard-Jones and electrostatic interactions related to the selected surface residues in the solute region are scaled in the replicas at higher solute temperatures, reducing inter-domain interactions for enhancing the domain motions. This treatment works nicely to avoid trapping the simulations at one of the local energy minima and allows them to explore more combinations of “possible” inter-domain interactions including salt-bridge interactions between two domains.

The other advantage in gREST_SSCR is that we can apply this scheme to very large biomolecular systems, when we focus on functionally important domain motions. The number of atoms in surface charged residues is still limited even in very large soluble proteins, membrane proteins or protein/nucleic acid complexes.

### 3.2. Molecular Mechanisms Underlying Ligand-Induced Conformational Changes of RBP

Using gREST_SSCR, we observed a smooth transition pathway in Holo RBP_G134R_ and a wider conformational space in Apo. In the smooth transition pathway in Holo, a non-linear correlation between hinge and twist angles is obtained in Figure 2. Since the intermediate structures in the pathway are largely different from both the closed and open forms, the non-native salt-bridge interactions between the two domains play important roles in the stabilization. We consider that the intermediate structures (H_CL_, H_OL_ and A_OL_) are meaningful, since they are also found with minor populations in 2 μs MD simulations starting from Holo and Apo forms. However, due to their transient nature, it is very difficult to detect them experimentally. To understand molecular mechanisms underlying the open-to-closed conformational transitions in RBP_G134R_, we may consider the possibilities of induced-fit or conformational selection [10,11,45,53]. We observe relatively larger conformational fluctuations in Apo and H_O_ in Holo, which may suggest the conformational selection mechanisms. However, the conformational space in Apo gREST_SSCR and cMD simulations cannot cover the X-ray structure of Holo closed form. H_C_ was sampled only in Holo simulation with a bound ribose. So, a pure conformational selection mechanism might not be applicable to this system. Since we added a restraint function between a bound ribose and Ser103 in all the Holo simulations, we cannot examine the effect of ligand binding in the conformational transitions in great detail. For this purpose, binding free-energy calculations in different metastable states is useful to give us more quantitative information of protein-ligand interactions.

### 3.3. General Applications of gREST and gREST_SSCR

The proposed method paves the way for further applications as well as the developments of other approaches within the framework of gREST. For instance, gREST_SSCR can be used for more efficient sampling of intrinsically disordered regions/proteins (IDR/IDP). Note that several IDRs’ sequences have high contents of charged residues. Beside small peptides, the significance reduction in the number of particles in the selected solute region allows for more applications in large multidomain/multichain proteins. However, we need to emphasize that gREST_SSCR is not the only choice to investigate a variety of conformational motions in any biomacromolecules. The original gREST contains the advantages of flexible selection of the solute region over the conventional REST/REST2 schemes. It is also worth testing the method to study folding/unfolding pathways in monomeric proteins. Wherein, another possible approach is the selection of buried hydrophobic residues to study protein folding in protein stabilized by hydrophobic cores. This choice is, in fact, almost the opposite approach to the current gREST_SSCR, while it seems reasonable for investigating folding-unfolding transitions of a small protein in solution which are driven by the hydrophobic interactions. In general, solute particles and potential energy term selections can be tailored to answer specific questions of interest in each study. We do not know the best solute selection in all cases, while running several short single-replica simulations at one high solute temperature is a good way to decide a reasonable solute selection in each case.

## 4. Materials and Methods

### 4.1. Modeling of RBPG134R for MD Simulations

The X-ray structure of Holo RBP_G134R_ in complex with a ribose (PDB:1drj) was used for the Holo simulations [44]. The chain A of wild type RBP in Apo (PDB:1urp) [47] was used as an initial structure, after mutating the 134th glycine to arginine for consistency with the Holo simulations. All histidine residues were kept neutral except for His152, which was predicted to be protonated based on the pKa calculations using the PROPKA 3.0 program and structural analysis [55]. Both RBP_G134R_ in Holo and Apo states were solvated in cubic boxes using CHARMM-GUI [56]. Wherein, the simulation system of Holo RBP_G134R_ consists of 54,523 atoms including 16,809 water molecules in a cubic box with a length of 81 Å. Similarly, the system of Apo RBP_G134R_ consists of 60,416 atoms including 18,780 water molecules in a cubic box with a length of 84.1 Å. Both systems were neutral, and no ions were added to simulation boxes.

### 4.2. cMD Simulations

Both cMD and gREST_SSCR simulations were performed using GENESIS software [57,58]. CHARMM36m, CHARMM carbohydrate force field and TIP3P models were used for RBP_G134R_, a ribose and water molecules, respectively [59,60,61]. First, both systems were energy minimized for 10,000 steps, applying positional restraints on backbone heavy atoms. Second, simulation boxes were heated up to 300.00 K in a step wise protocol within 100 ps using leap-frog integrator and Langevin thermostat [62,63], maintaining the same restraints. Third, the systems were equilibrated in the NPT ensemble at 300.00 K and 1 bar using the Bussi thermostat/barostat [64] and velocity Verlet integrator for 1 ns with a time step of 2 fs [65,66]. Fourth, the final equilibration steps in NPT were performed for 2 ns using the Bussi thermostat/barostat and the RESPA multiple time-step integrator [67] with a fast motion time step of 2.5 fs. The slow motion, which is based on the reciprocal interaction of particle mesh Ewald (PME) [68], was computed every other step. Finally, cMD production runs were performed for 2 μs in the NVT ensemble using the Bussi thermostat and RESPA integrator with the time step of the previous step. The whole simulation trajectory was analyzed. A distance restraint between the center of mass (COM) of a ribose and the Cα atom of Ser103 in Holo RBP_G134R_ was applied using a force constant of 10 kcal mol^−1^ Å^−2^ to prevent the substrate from releasing during the simulations. Water molecules and bonds involving hydrogens were constrained with SETTLE and SHAKE [69,70], respectively. Long-range electrostatic interaction was calculated using PME. Lennard-Jones interactions were smoothly reduced to zero from 10 to 12 Å using a switching function.

### 4.3. gREST_SSCR Simulations

gREST_SSCR simulations were performed using 12 replicas including the following solute temperatures, 300.00, 318.11, 337.11, 357.10, 378.07, 400.11, 423.25, 447.60, 473.14, 499.97, 528.13 and 550.00 K. The solvent temperatures were all kept at 300.00 K. Before the production run, all the replicas were further equilibrated for 1 ns, where no exchanges were allowed. Production runs were performed for 250 ns per replica in the NVT ensemble using the Bussi thermostat and the RESPA integrator with a fast motion time step of 2.5 fs. The slow motion, which is based on the reciprocal interaction of particle mesh Ewald (PME), was computed every other step. In gREST_SSCR, replica exchanges were tried every 5000 steps. Structures were saved at every 1000 steps. The solute region consists of: (1) positive charged residues: R90, K110, K118, R134, R139, R141, R166, K168, K243, K260 and K266; (2) negative charged residues: D67, D69, D89, D104, E140, D163, E192, D215, D219, E221 and D264. These residues were selected in the three criteria: (i) All the surface charged residues near the interface of the two domains are selected if their Cα atoms are located less than 6 Å away from the Cα atoms of the nearest interface residues. (ii) The residues in the hinge region between the two rigid domains (D104, K260, D264, and K266). (iii) A few more surface charged residues away from the interfaces are added to keep the charge neutrality in the solute region (K110, 118, and 243). One can use the SASA values for the amino-acid residues to distinct the surface residues or not. In the current case, the average values (closed and open) in the selected charged surface residues are distributed between 247.0 and 357.4 Å^2^. Only Lennard-Jones (LJ) and electrostatic terms of these residues were chosen in the solute region. We have also tested other selection criteria such as the connecting loops’ residues with dihedral terms. However, the combination of LJ and electrostatic interactions of surface charged residues in the two domains were found to outperform other criteria. The calculated replica exchange probabilities were between 15% and 46% with an average of 27% and 24% in the Holo and Apo simulations, respectively.

gREST_SSCR is a subset of the gREST method, which allows us to select the solute region in several ways. Equation (1) represents the modified potential energy of gREST for the replica *a* at solute temperature index *m*:

(1) EmgREST,a= βmβ0EuuXa + ∑iβmβ0kili Euv,iXa + EvvXa

The first and second terms represent solute-solute (*uu)* and solute-solvent (*uv)* interactions, respectively. While the third term represents solvent-solvent (*vv)* interaction. $\beta_{m}$ and $\beta_{0}$ represent solute and solvent temperatures, respectively. *l_i_* and *k_i_* are the maximum number of atoms and number of solute atoms that form solute-solvent interaction. *l_i_* and *k_i_* for the Coulomb and LJ interactions are 2 and 1, respectively.

### 4.4. Simulation Trajectory Analysis

All trajectories were analyzed using the GENESIS analysis tools. The VMD program was used for trajectories visualization and snapshot structures generation [71]. The structure figures in this paper were prepared using the PyMOL program [72]. The twist angle was calculated using COMs of NTD and CTD as well as the two COMs of these residues: (1) 99–100, 236–237 and 258–259 and (2) 108–109, 230–231 and 269–270. These residues represent the bases of NTD and CTD, respectively. Residue-residue contact distance maps in Figure S4 were calculated using the iTrajComp VMD plugin. Based on the average distances, 41 potential salt bridges were identified, and hydrogen bond (H-bond) analysis was performed to determine important interactions in each cluster. The *K*-mean algorithm in GENESIS software was used to classify the obtained conformations in gREST_SSCR simulations at 300.00 K. Wherein the number of clusters were determined based on the number of observed metastable states in the hinge/twist free energy maps (Figure 2d). All Cα atoms were included as a criterion for clustering. Consequently, Holo simulations were clustered into four main clusters: Holo_closed (H_C_), Holo_closed_like (H_CL_), Holo_open_like (H_OL_) and Holo_open (H_O_) conformations (Figure S5a). H_C,_ H_CL_, H_OL_ and H_O_ constitute 40.2%, 14.1%, 10.5% and 35.2% of the conformations at 300 K. In H_O,_ all conformations with very large hinge angles (>146°) were excluded from the analysis. The center of each cluster is shown in Figure 2d. Similarly, gREST_SSCR simulation of Apo RBP_G134R_ was clustered into two clusters Apo_open (A_O_) and Apo_open_like (A_OL_) where they represent 72.0% and 28.0% of the snapshots, respectively (Figure S5b). In cMD Holo simulation, Holo_closed (H_C_) conformations were selected as the first 875 ns of the simulation, based on the RMSD of the Cα atoms from the X-ray structure. In cMD Apo simulation, A_T_ structures were characterized as any conformation with twist angles less than 58°. The number of conformations in A_T_ is minor, representing 0.2% of the simulation.

## 5. Conclusions

We propose an atomistic MD approach to investigate large-scale conformational transitions in multidomain proteins. Wherein Lennard-Jones and electrostatic terms of selected surface charged residues are used as the solute region in gREST simulations. This approach, gREST_SSCR, is applied to the open-to-closed transitions in the G134R mutant of RBP, RBP_G134R_. The simulations do not affect intra-domain stability but enhance relative domain motions in both states by preventing strong inter-domain H-bonds at higher solute temperatures. In Apo state, which takes various open structures, gREST_SSCR can sample two stable conformers, although a single basin with a wide distribution is obtained in cMD at the same temperature. The sampling space in Holo via gREST_SSCR is extended significantly compared to that with cMD. gREST_SSCR can sample four representative conformers: two are similar to the open and closed X-ray structures, while the other two intermediates are newly discovered. They are stabilized via non-native salt-bridge (electrostatic) interactions, which are not accessible by cMD and experimental measurements. gREST_SSCR is a simple but promising approach to investigate large-scale domain motion in various biological systems including very large proteins and protein/nucleic acids complexes.

## Figures and Tables

**Table 1:** Percentages of hydrogen bonding for salt bridge interactions in each metastable state in the simulations. Dominant interactions are highlighted with bold font. * represents salt bridge interactions in the X-ray structure of Holo RBP_G134R_ (PDB:1drj). In cMD, H_C_ represents the first 875 ns of the Holo simulation, while A_T_ represents Apo conformations with the twist angle of less 58°. The percentages of hydrogen bonding larger than 30% is in the bold fonts.

Method (State)		gREST_SSCR (Holo)				gREST_SSCR (Apo)		cMD (Holo)		cMD (Apo)	
Residue (domain)	Residue (domain)	H_C_	H_CL_	H_OL_	H_O_	A_OL_	A_O_	All	H_C_	All	A_T_
Asp67 * (NTD)	Arg134 * (CTD)	**82.8** ± 1.4	5.0 ± 0.6	0	0	0	0	**47.7** ± 3.8	**79.8** ± 1.1	<0.1	0
Asp69 * (NTD)	Arg134 * (CTD)	19.4 ± 1.3	**78.0** ± 2.4	**30.2** ± 4.5	<0.4	<0.1	<0.1	19.0 ± 1.8	16.0 ± 0.8	<0.1	0
Asp69 (NTD)	Arg139 (CTD)	23.5 ± 1.0	<0.5	0	0	0.1	0	18.0 ± 2.5	**39.7** ± 2.4	0	<0.1
Arg90 (NTD)	Glu140 (CTD)	0	0	0	0	**86.8** ± 0.9	<1.1	11.8 ± 3.0	0	1.8 ± 0.3	**74.5**
Arg90 (NTD)	Asp215 (CTD)	0	0	<0.5	**34.8** ± 4.2	0	**37.2** ± 3.9	0	0	**30.9** ± 2.3	0
Glu140 (CTD)	Lys260 (Hinge)	3.9 ± 0.3	**44.5** ± 3.8	**80.4** ± 1.2	**79.2** ± 1.4	<0.7	**59.0** ± 3.5	6.7 ± 1.3	3.6 ± 0.1	**62.4** ± 1.1	0
Glu221 (CTD)	Lys266 (Hinge)	24.0 ± 1.2	26.9 ± 1.1	<0.2	0	<0.6	<0.3	19.4 ± 1.8	**31.2** ± 0.8	<0.3	0

## Data Availability

The data presented in this study are available on request from the corresponding author. The data are not publicly available due to their large size.

## Supplementary Materials

Supplementary materials can be found at https://www.mdpi.com/1422-0067/22/1/270/s1.

## Abbreviations

MD	Molecular dynamics
gREST	Generalized replica exchange with solute tempering
gREST_SSCR	gREST selected surface charged residues
RBP	Ribose binding protein
NTD	N-terminal domain
CTD	C-terminal domain
Rg	Radius of gyration
RMSD	Root mean square deviation
