# Supplementary material for: Exploring Large Domain Motions in Proteins Using Atomistic Molecular Dynamics with Enhanced Conformational Sampling
Source: Int J Mol Sci. 2020 Dec 29;22(1):270. doi: 10.3390/ijms22010270 (PMC7796230; doi:10.3390/ijms22010270)
Supplement: Supplementary file 1 [file ijms-22-00270-s001.pdf]

Supporting Information of

Exploring large domain motions in proteins using atomistic molecular dynamics with enhanced conformational sampling

Hisham M Dokainish [1] and Yuji Sugita [1, 2, 3, *]

[1] RIKEN Cluster for Pioneering Research, 2-1 Hirosawa, Wako, Saitama 351-0198, Japan; hisham.dokainish@riken.jp, sugita@riken.jp

[2] RIKEN Center for Computational Science, Integrated Innovation Building 7F, 6-7-1 Minatojima-minamimachi, Chuo-ku, Kobe, Hyogo 650-0047, Japan

[3] RIKEN Center for Biosystems Dynamics Research, Integrated Innovation Building 7F, 6-7-1 Minatojima-minamimachi, Chuo-ku, Kobe, Hyogo 650-0047, Japan

* Correspondence: sugita@riken.jp; Tel.: +81-48-462-1407 (Y.S.)

Supporting Figures

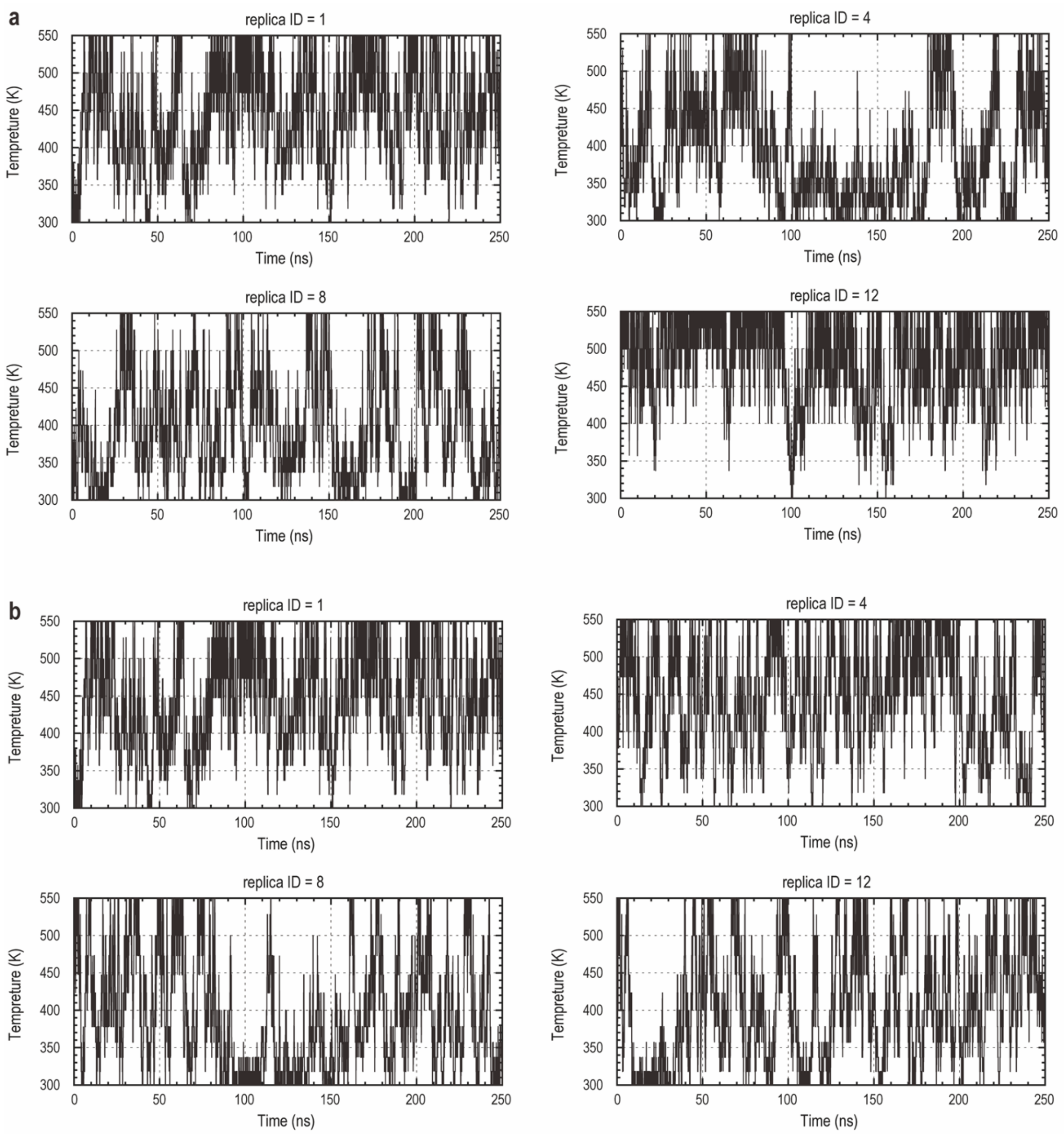

Figure S1. Time series of solute temperature index along the selected replicas (1, 4, 8, and 12) in Holo (a) and Apo (b) gREST_SSCR simulations.

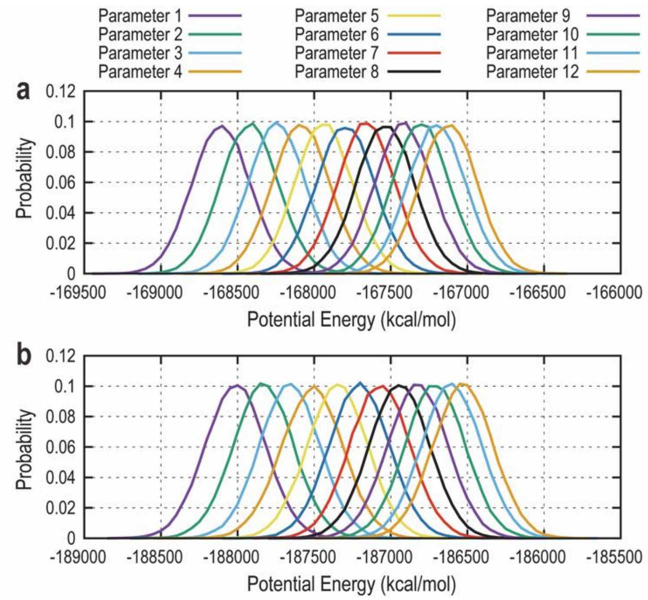

Figure S2. Probability distributions of modified potential energies in gREST_SSCR simulations. Those at 12 solute temperatures in Holo (a) and Apo (b) gREST_SSCR simulations are shown.

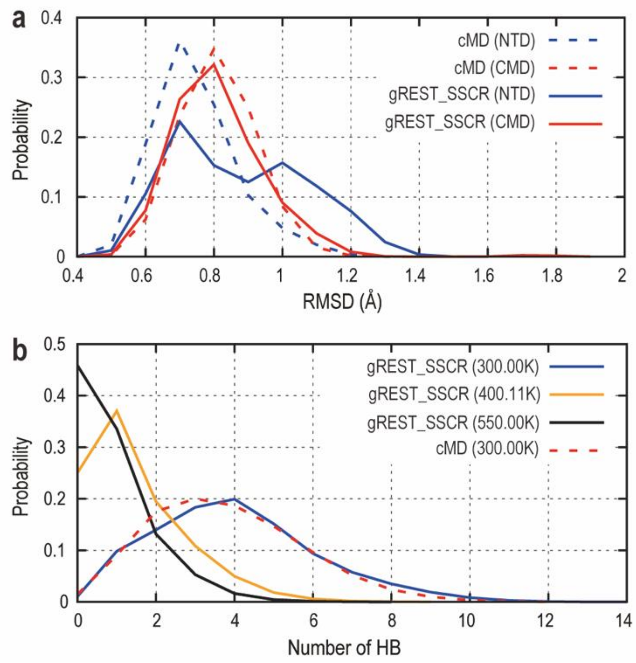

Figure S3. (a) Probability distributions of the Cα atoms RMSD of NTD and CTD in cMD (dashed line) and gREST_SSCR Apo simulation (solid line) both at 300.00 K. RMSD of NTD and CTD are shown in blue and red, respectively. (b) Probability distributions of H-bonds in the Apo state between the 22 selected residues in the solute region of gREST_SSCR simulation at 300.00, 400.11, and 550.00 K (solute temperatures). As a reference, the same distribution obtained in cMD at 300.00 K is shown as a dotted line.

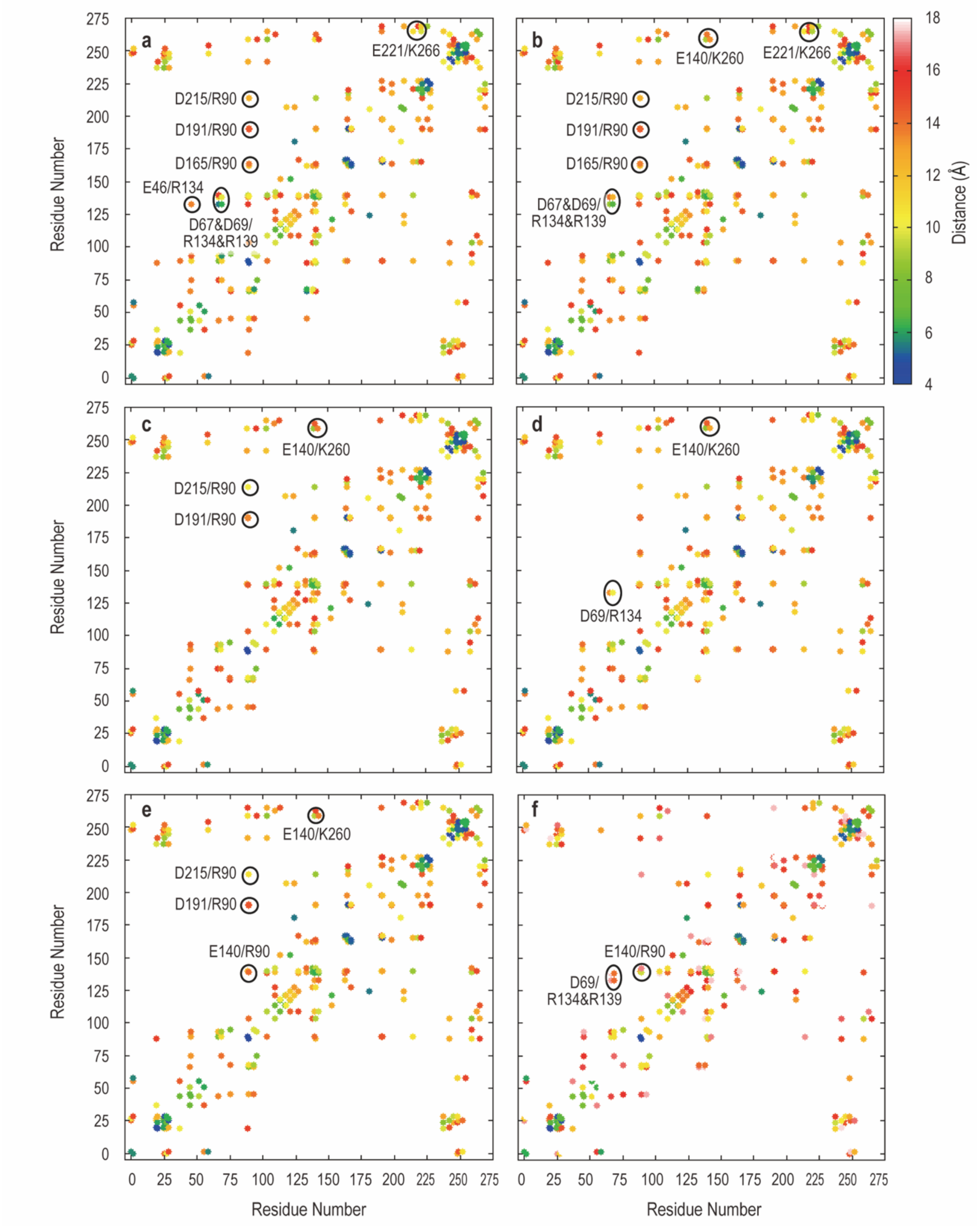

Figure S4. Residue-residue average salt-bridge distances of six metastable forms, (a) H_C, (b) H_{CL}, (c) H_O, (d) H_{OL}, (e) A_O, and (f) A_{OL}. Circled interactions are inter-domain salt-bridges listed in Table 1.

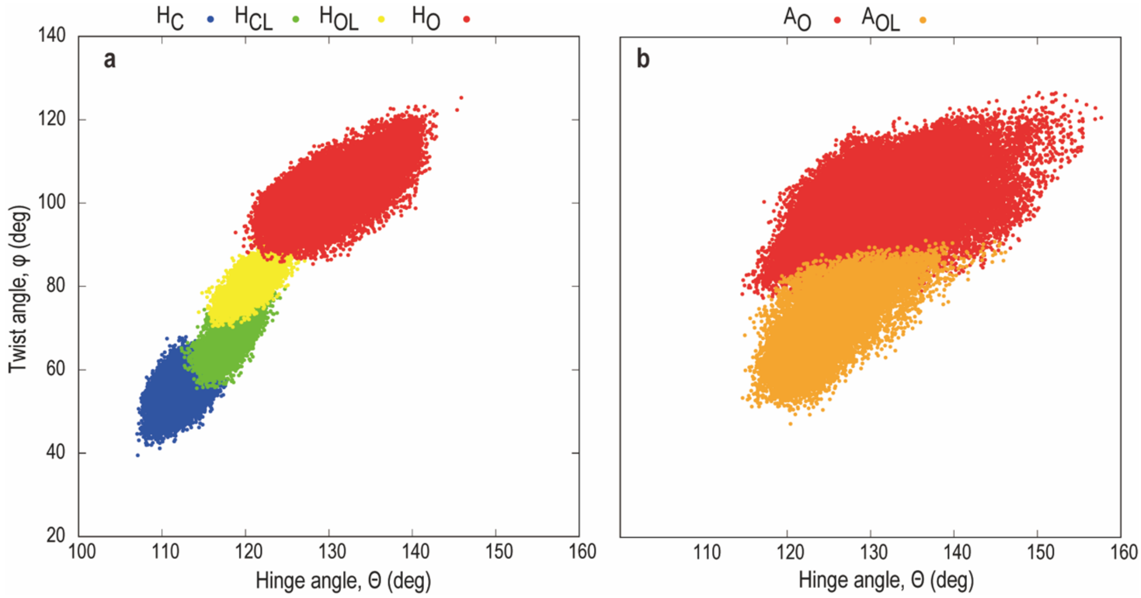

Figure S5. Structural clustering of gREST_SSCR Holo (a) and Apo (b) simulation trajectories in the Hinge and Twist angle conformational space. In a, H_C, H_{CL}, H_{OL} and H_O conformations are shown in blue, green, yellow and red, respectively. In b. A_O and A_{OL} are shown in red and orange, respectively.
